# Abundant Atribacteria in deep marine sediment from the Adélie Basin, Antarctica

**DOI:** 10.3389/fmicb.2015.00872

**Published:** 2015-08-26

**Authors:** Stephanie A. Carr, Beth N. Orcutt, Kevin W. Mandernack, John R. Spear

**Affiliations:** ^1^Department of Civil and Environmental Engineering, Colorado School of Mines, GoldenCO, USA; ^2^Bigelow Laboratory for Ocean Sciences, East BoothbayME, USA; ^3^Department of Earth Sciences, Indiana University – Purdue University Indianapolis, IndianapolisIN, USA

**Keywords:** geomicrobiology, methane, sediment, Antarctica, single cell genomics, Atribacteria

## Abstract

Bacteria belonging to the newly classified candidate phylum “Atribacteria” (formerly referred to as “OP9” and “JS1”) are common in anoxic methane-rich sediments. However, the metabolic functions and biogeochemical role of these microorganisms in the subsurface remains unrealized due to the lack of pure culture representatives. In this study of deep sediment from Antarctica’s Adélie Basin, collected during Expedition 318 of the Integrated Ocean Drilling Program (IODP), Atribacteria-related sequences of the 16S rRNA gene were abundant (up to 51% of the sequences) and steadily increased in relative abundance with depth throughout the methane-rich zones. To better understand the metabolic potential of Atribacteria within this environment, and to compare with phylogenetically distinct Atribacteria from non-deep-sea environments, individual cells were sorted for single cell genomics from sediment collected from 97.41 m below the seafloor from IODP Hole U1357C. As observed for non-marine Atribacteria, a partial single cell genome suggests a heterotrophic metabolism, with Atribacteria potentially producing fermentation products such as acetate, ethanol, and CO_2_. These products may in turn support methanogens within the sediment microbial community and explain the frequent occurrence of Atribacteria in anoxic methane-rich sediments. This first report of a single cell genome from deep sediment broadens the known diversity within the Atribacteria phylum and highlights the potential role of Atribacteria in carbon cycling in deep sediment.

## Introduction

Particles fall to the ocean floor from diverse sources at various rates; consequently, available carbon substrates, and redox conditions differ with geographical locations (Jahnke and Jackson, 1992; Jahnke, 1996). Within this spectrum of subseafloor conditions, microbial biomass is estimated to be as great as 3 × 10^29^ cells—similar in magnitude to all life in the ocean water (Kallmeyer et al., 2012) —yet the diversity of metabolic potentials within this environment is largely unexplored due to the particular lack of microbial cultures obtained from subsurface environments. Importantly, global estimates of microbial biomass and metabolic potential in subsurface sediment lack sufficient baseline data, particularly from polar regions where sampling opportunities are very limited. Therefore, it is unclear if diversity trends or microorganisms observed in non-polar regions are also found at higher latitudes. To this end, this study sought to investigate the diversity and metabolic functions of microbial communities in deep sediment of the Adélie Basin, a deep bay located 100 km offshore of Antarctica’s Wilkes Land Margin.

The Wilkes Land Margin was targeted by Expedition 318 of the Integrated Ocean Drilling Program (IODP) in 2010 (Expedition 318 Scientists, 2011a). Here, continental and algal detritus fall to the seafloor at a relatively high rate of ∼2 cm yr^-1^, providing copious organic carbon that is respired by sediment microorganisms, leading to anoxic conditions (Expedition 318 Scientists, 2011). Sulfate concentrations within this basin are quickly depleted within the first 2 m of the sediment–water interface. Methane concentrations within this setting reach a maximal concentration of 12.8 mM at 22 m below seafloor (mbsf; Expedition 318 Scientists, 2011), suggesting active methanogenesis and a narrow sulfate-methane transition zone. This geochemical framework is similar to other near-shore, organic-carbon rich, marine sediment environments (D’Hondt et al., 2002; Inagaki et al., 2006; Webster et al., 2006a) providing an opportunity to compare sediment microbial communities from this Antarctic site to those observed previously.

One common microbial group from near-shore, organic carbon replete, methane-rich marine sediment is the Atribacteria, a recently classified phylum previously referred to as OP9 and JS1 (Rochelle et al., 1992; Hugenholtz et al., 1998; Rinke et al., 2013). Atribacteria have primarily been identified through sequencing of the 16S rRNA gene from a number of anoxic sedimentary environments including tidal flats (Wilms et al., 2006; Webster et al., 2007), brackish sediments (Webster et al., 2004; Rinke et al., 2013), mud volcanoes (Niemann et al., 2006), hydrothermal areas (Teske et al., 2002), organic rich deep-sea sediment (Webster et al., 2006a), and the hydrate-bearing sediments of the Nankai Trough, the Sea of Okhotsk, and the Peru and Cascadia margins (Reed et al., 2002; Inagaki et al., 2003, 2006; Newberry et al., 2004). Of these sites, those associated with methane hydrates have higher relative abundances of Atribacteria sequences, generally over 50% of sequences (Inagaki et al., 2003, 2006; Newberry et al., 2004), suggesting a selection for Atribacteria in methane-rich sediment (Inagaki et al., 2006). The biogeochemical role(s) of this group within anoxic sediment environments remains unclear given the lack of pure isolates; however, isotope enrichment studies of marine sediment documented the incorporation of ^13^C-labeled acetate and glucose into Atribacteria DNA and suggest a heterotrophic metabolism (Webster et al., 2006b).

Atribacteria have also been identified in non-marine settings such as terrestrial hot springs and anaerobic sludge bioreactors (Chouari et al., 2005; Dodsworth et al., 2013). Single cell genomic (Dodsworth et al., 2013; Rinke et al., 2013) and metagenomic (Dodsworth et al., 2013) approaches have been applied to some of these non-marine samples to determine the genetic potential of this group. For example, two candidate Atribacteria species—*Candidatus Caldatribacterium californiense* and *Ca. Caldatribateriam saccharofermentans*—were identified from geothermal hot springs (Dodsworth et al., 2013). Both are hypothesized to produce hydrogen, acetate, and ethanol as a result of sugar fermentation, further supporting the inference of a heterotrophic lifestyle from marine enrichments (Webster et al., 2006b).

Considering that Atribacteria are known to be abundant in organic rich marine sediment, that fermentative lifestyles are suggested from genomic studies of non-marine Atribacteria, and that fermentation is a likely process in the anoxic sediment of the Adélie Basin, this study provided a natural laboratory for exploring the possible role of Atribacteria in marine sediment biogeochemistry. We first examined the sequence abundance of this group in Adélie Basin sediments in the context of sediment biogeochemical processes. We then applied single cell genomic approaches (Stepanauskas and Sieracki, 2007; Rinke et al., 2013; Lasken and McLean, 2014) to explore the genetic potential of this group, leveraging off recently developed techniques used to examine uncultivated microorganisms from shallow (<8 mbsf) sediment (Lloyd et al., 2013; Kaster et al., 2014; Wasmund et al., 2014). Focusing on a deep sediment sample (97.41 mbsf) with a high relative abundance of Atribacteria (based on 16S rRNA gene pyrotag sequencing), we provide the first microbial data from the deep biosphere of a remote and highly productive region off Antarctica, the first partial genome from deep subsurface sediment, and the first partial genome of a marine Atribacteria cell. The work presented here investigates the metabolic potential of Atribacteria, and the potential involvement of this group in carbon cycling in marine sediment.

## Materials and Methods

### Sample Collection

**FIGURE 1:**
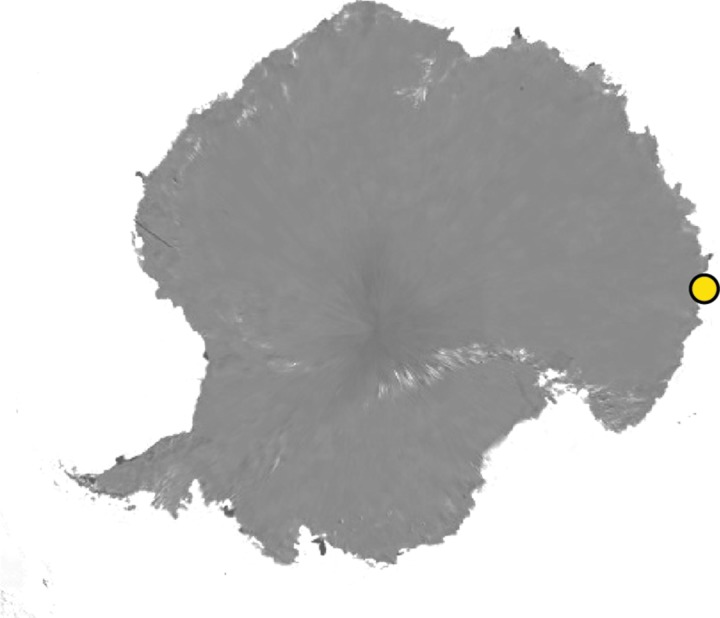
**Location of IODP Site U1357 in the Adélie Basin offshore Antarctica (Antarctic Image Source: “Antarctica” 90°S, and 0°E. Google Earth, December 13, 1998, accessed December 1, 2014)**.

Sediment of the Adélie Basin was sampled during IODP Expedition 318 in 2010 from borehole U1357C, which is described in further detail elsewhere (Expedition 318 Scientists, 2011a). Briefly, Site U1357 is located directly offshore of the Adélie Drift along the Wilkes Land Margin (66°24.8′S, 140°25.5′E, **Figure 1**) at roughly 1000 m water depth. The recovered sediment consisted primarily of diatom ooze (80–99%) with occasional fine layers of clay and silt in alternating greenish brown and olive green varves representing annual algal blooms. High annual primary productivity in surface ocean waters coupled with terrestrial runoff from the Antarctic mainland leads to relatively high sedimentation rates of 2 cm yr^-1^ (Expedition 318 Scientists, 2011).

Samples for molecular biological analysis were collected shipboard as described in detail elsewhere (Expedition 318 Scientists, 2011b). Briefly, 10 cm sections of the intact core were taken for interstitial water geochemistry and molecular analysis at varying depth resolution throughout the core. For molecular analyses, sterile cut-end 5 mL syringes were used to collect sediment from the center of core sections; these were immediately frozen at –80°C and maintained at this temperature during transport and storage to the home laboratory. While contamination tracers such as microspheres or perfluorocarbons (Lever et al., 2006) were not used during this expedition, samples collected from the ends of each core had anomalously high sulfate concentrations (a rough measure of contamination from seawater intrusion into sediment). DNA extracts from these samples (20 of 60 samples collected) were not analyzed.

**FIGURE 2:**
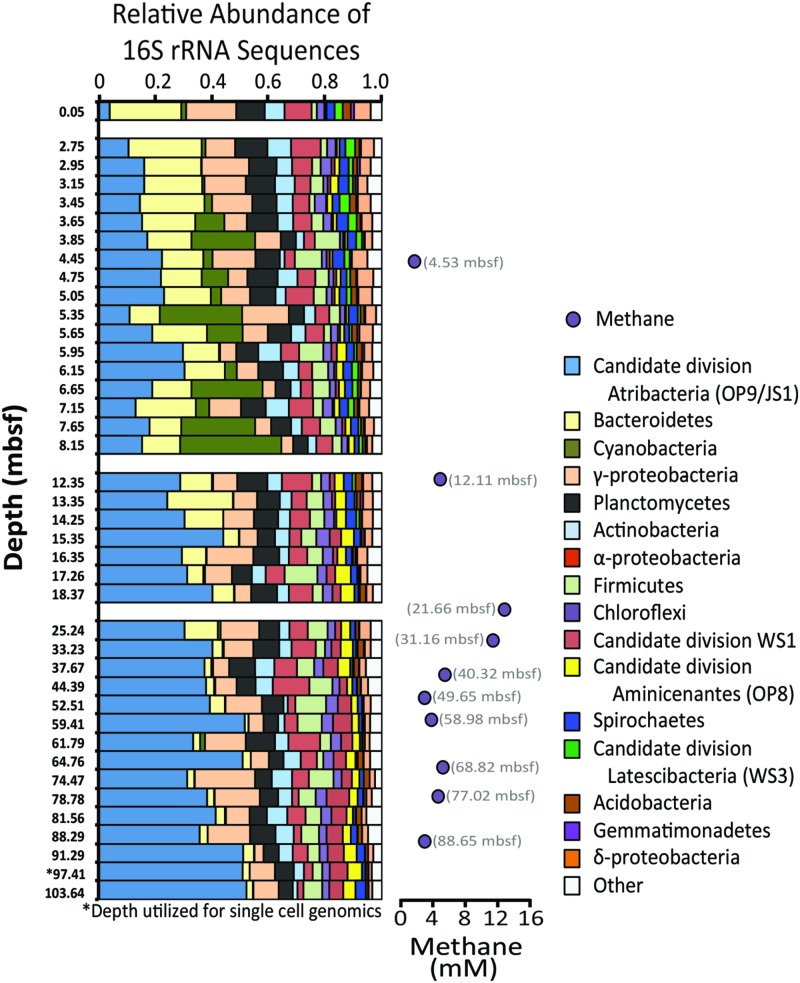
**Relative abundances of 16S rRNA gene sequences from bacterial phyla and subphyla detected from sediment depths indicated on the left side of panel.** Bacterial phyla representing less than 1% of operational taxonomic units (OTUs, defined as 97% or greater sequence similarity) in 90% of the samples were grouped as “other.” Methane concentrations (mM) are provided to the right. Given that the depth profile is not to scale, methane sample depths are provided.

Sediment geochemical data such as total organic carbon, dissolved inorganic carbon, methane, and sulfate concentrations were reported previously (Expedition 318 Scientists, 2011). To summarized briefly, concentrations of total organic carbon within the top 100 m of the core average 1.6 ± 0.2% weight percent, which is similar in concentration to other near-shore sediment of similar depth (Jahnke, 1996). Dissolved inorganic carbon was relatively high, increasing from 40 to 80 mM within the upper 20 m of sediment (deeper values are not available), indicating high organic carbon remineralization. Methane concentrations peaked at 12.8 mM at 21.61 mbsf, with a secondary peak of 5.2 mM at 68.79 mbsf (**Figure 2**). Sulfate was quickly depleted to below detection limit (10 μM, data not shown) within the first two meters of the sediment–water interface.

### DNA Extractions

Genomic DNA was extracted from sediment samples using a bead beating/phenol chloroform protocol modified from Zhou et al. (1996). Briefly, 0.1–1.0 g of sediment was diluted with 2 mL of lysis buffer (100 mM Tris [pH 8.0], 100 mM ethylenediaminetetraacetic acid (EDTA), 100 mM NaH_2_PO_4_, 1.5 M NaCl, 1% [w/v] cetrimonium bromide) and homogenized on a mini beadbeater (Biospec Products, Bartlesville, OK, USA) with 0.1 mm zircon beads for 30 s at the homogenize setting. Fifty microliter of a proteinase K solution (20 mg/ml) was added and samples were shaken at 150 rpm at 37°C for 30 min. Subsequently, 10 mL of 20% [w/v] sodium dodecyl sulfate (SDS) solution was added, and samples were incubated at 65°C for 2 h with inversions every 15 min. Samples were centrifuged at 6,000 × *g* for 10 min, and supernatants were decanted into clean microcentrifuge tubes. The remaining sediment was extracted a second time by adding an additional 1 mL of the lysis buffer and 10 mL of SDS, vortexing, incubating at 65°C for 10 min, centrifuging as above, and then combining the supernatants. Nucleic acids were extracted from the supernatants with an equal volume of phenol:chloroform:isoamyl alcohol (25:24:1 [v/v/v]; pH 8.0,10 mM Tris, 1 mM EDTA) followed by a second extraction with an equal volume of chloroform:isoamyl alcohol (24:1 [v/v]). Nucleic acids were precipitated with an equal volume of iced isopropanol and 10% [v/v] 3 M sodium acetate, followed by two ice cold 70% ethanol washes. Precipitates were brown in color with suspected humics and were purified using an ethidium bromide high salt extraction (Stemmer, 1991). Nucleic acids were resuspended in nuclease free water. Samples and extraction blanks were screened for amplifiable DNA by polymerase chain reaction (PCR) with PCR Master Mix (Promega #M7502) and the modified universal primers 515f and 927r of Osburn et al. (2011) using the following reaction conditions: 94°C denaturation for 2 min; 30 cycles of 95°C for 30 s, 55°C for 30 s, and 72°C for 45 s; and a final elongation step at 72°C for 5 min PCR product was then loaded onto an agarose gel to check for product bands with ethidium bromide staining. All extraction blanks yielded negative results.

### Pyrosequencing and Bioinformatic Processing

Amplicons of the 16S rRNA gene were prepared for 454 pyrosequencing using a PCR-touchdown annealing temperature strategy from Don et al. (1991) with procedure modifications and modified universal primers 515f and 927r of Osburn et al. (2011). These modified primers were selected based on improved coverage of the V4 and V5 region of the 16S rRNA gene (Osburn et al., 2011). According to the Silva TestProbe 3.0, forward and reverse primers covered 79.8 and 75.4% of archaea and 79.1 and 76.1% of bacteria with no mismatches, respectively (Pruesse et al., 2007). Forward primers included the 454 Life Science A adaptors and a sample specific 8 nt barcode. The reverse primer included the 454 Life Science B adaptors. Amplicon concentrations for each sample were quantified using a 2100 Bioanalyzer (Agilent Technologies, Colorado Springs, CO, USA), pooled (22 ng DNA/sample) and concentrated using a Savant DNA 12 Speed Vac Concentrator (Thermo Scientific, Waltham, MA, USA). The pooled DNA was gel purified using the Montage DNA Gel Extraction Kit (Millipore, Bellerica, MA, USA), and sequenced on a Roche 454 FLX titanium platform at Engencore, University of South Carolina (now Selah Genomics).

Pyrosequencing reads were analyzed using the Quantitative Insights Into Microbial Ecology (QIIME) Pipeline (Caporaso et al., 2010). Reads between 200 and 500 base pairs with a quality score of 27 or above were denoised using the QIIME denoiser for titanium runs, and clustered into operational taxonomic units (OTUs) at a 97% similarity threshold using UCLUST (Edgar, 2010). Taxonomy of OTUs was assigned by BLAST (Altschul et al., 1990) against the Silva SSU NR Reference database, release 102 (Pruesse et al., 2007) within QIIME. Chimeras were identified and removed using QIIME’s ChimeraSlayer Wrapper. Extraction replicates demonstrated consistent community results (Supplementary Figure S1), with Pearson Correlation coefficients > 0.96. Sequences were deposited in the MG-RAST database under accession numbers 4624791.3–4634830.3.

### Single Cell Sorting, Genome Amplification, Sequencing, and Annotation

Sorting of individual cells from unfixed frozen sediment from 97.41 mbsf was attempted for single cell genomics using the Single Cell Genomics Center (SCGC) at the Bigelow Laboratory for Ocean Sciences. While the sediment was not preserved with recommended fixatives to prevent cell lysis during storage (Swan et al., 2011; Stepanauskas, 2012; Lloyd et al., 2013), and although the samples had gone through at least one round of freeze-thaw for bulk DNA extraction which may have introduced cell lysis, the high relative abundance of target microorganisms in the sample suggested this approach might still be successful in recovering intact cells. Approximately 0.5 g of frozen sediment was diluted in 1 mL of filter-sterilized seawater and vortexed for 30 s to liberate cells from the sediment matrix, modifying methods developed previously (Lloyd et al., 2013). Sediment was then separated from cells by gentle centrifugation at 2000 rpm for 30 s The cell suspension was treated with SYTO-9 DNA stain and sorted into two 384-well plates using SCGC’s standard pipeline. Both sorted plates were subjected to physical lysis treatments (five freeze-thaw cycles), and the second plate also experienced an alkaline lysis treatment (Raghunathan et al., 2005). DNA amplification by multiple displacement amplification was performed according to published protocols (Swan et al., 2011). A total of eight amplification reactions had successful DNA amplification from the two plates, but only one of these reactions had a positive screen for Atribacteria with bacterial specific 16S rRNA gene primers (27F and 907R; Lane, 1991). This low recovery rate is likely a result of not fixing the samples to prevent cell lysis and/or different susceptibility of deep sediment cells to cell lysis.

The initial assembly of Atribacteria bacterium SCGC AD-561-N23 is publically available within the IMG system (taxon ID 2588254308) and the sequence for the 16S rRNA gene is available within the IMG system and Supplementary Materials. A detailed assembly procedure can be downloaded from the QC.finalReport.pdf available at http://genome.jgi.doe.gov/CandivSCAD561N23/CandivSCAD561N23.download.html. Briefly, single-cell amplified genomic (SAG) DNA was sequenced, assembled and annotated at the United States Department of Energy’s Joint Genome Institute (JGI) following their standard pipeline for Illumina HiSeq 2000 platform sequencing. Illumina reads were screened using JGI’s in-house DUK filtering program (Mingkun et al., unpublished). Trimmed reads were assembled using SPAdes (version 3.0.0) with the following parameters (–t 8 –m 40 – –sc – –careful – –12; Bankevich et al., 2012). Once released to Integrated Microbial Genomes (IMG) system, manual screening and removal of potential contaminate sequences according to JGI’s single cell data decontamination protocol (Clingenpeel, 2015). Scaffolds with GC contents that varied from the genome average more than 10% and clustered as a distinct group according to a kmer analysis (IMG, fragment window 5000 bp, fragment step 500 bp, oligomer size 5, minimum variation 10) were identified as potential contaminates and were removed from the *de novo* assembly (with the exception of scaffolds that contained ribosomal DNA). This screened genome was submitted to the IMG database as GOLD project Gp0087948, titled “Candidate division JS1 bacterium SCGC AD-560-N23 (manually screened).”

Gene annotations were performed using both IMG and the Rapid Annotation using Subsystem Technology (RAST) platforms (Aziz et al., 2008; Overbeek et al., 2013; Markowitz et al., 2014). Discrepancies between annotations were investigated by comparing coding sequences of genes against GenBank non-redundant protein sequence and Swiss-Prot Databases by BLASTP (Altschul et al., 1990). Genome completeness was estimated by comparing the annotated genome sequence against a list of conserved single copy bacterial genes (Rinke et al., 2013) using HMMER3 software (Eddy, 2011). Average nucleotide identify (ANI) comparisons between the one single cell genome from this study and publically available genomes from Atribacteria (**Table 1**) were calculated using JSpecies software (Richter and Rosselló-Móra, 2009) and standard parameters determined elsewhere (Goris et al., 2007).

**Table 1:** Genome characteristics of single amplified genomes (SAGs) from Atribacteria, including sample location and GC content.

SAG Name	Environment	GC (%) content	Genome size (Kbp)	ANI^∗^ (%)	Genome alignment	Reference
Atribacteria bacterium SCGC AD-561-N23	Marine sediment	38	209	–		This study
Atribacteria bacterium SCGC AAA252-M02	Brackish lake	35	2,091	87	33%	I
Atribacteria bacterium JGI 0000014-F07	Lagoon sediment	35	321	77	2%	I
Atribacteria bacterium JGI 0000079-L04	Terephthalate degrading bioreactor	36	1,011	68	14%	I
Atribacteria bacterium JGI 0000079-F20	Terephthalate degrading bioreactor	37	719	67	<1%	I
*Candidatus Caldatribacterium californiense* OP9-cSCG	Hot spring	55	2,080	59	3%	II

*^∗^Average nucleotide identity comparison between Antarctic bacterium SCGC AD-561-N23 and the given SAG and the percentage of I. Rinke et al., 2013; II. Dodsworth et al., 2013.*

### Phylogenetic Tree Construction

Operational taxonomic units identified from pyrosequencing and the 16S rRNA gene sequences for the one single cell genome were phylogenetically compared to an Atribacteria (i.e., OP9 and JS1) reference tree. The reference tree was reconstructed using the same 204 Atribacteria sequences and 11 Gemmatimonadetes sequences utilized by Yarza et al. (2014) and five publicly available Atribacteria genomes (**Table 1**). Sequence alignments were constructed using SSU-align and regions of low posterior probabilities were masked out using SSU-mask (Nawrocki et al., 2009). A phylogenetic tree was constructed using RaxML version 7.2.7 with the rapid bootstrapping algorithm and GTRGAMMA as the nucleotide substitution model and evolutionary mode (Stamatakis et al., 2008). Bootstrap values were calculated from 1000 bootstrap inferences. The 16S rRNA gene sequences from Atribacteria bacterium SCGC AD-561-N28, OTUs 613, and 2153 and a closely related short (<1200 bp) Atribacteria sequences from Antarctic settings (AF147496) were aligned using the same filter mask developed for the reference tree alignment (Altschul et al., 1990; Desantis et al., 2006). Aligned sequences were then placed into the constructed phylogenic tree using pplacer (Matsen et al., 2010).

## Results and Discussion

### Microbial Community Composition of Deep Adélie Basin Sediment

Pyrosequencing of the 16S rRNA gene was performed at 40 sample depths (0.05–104 mbsf) to evaluate changes of the microbial diversity with depth in Antarctic sediment. After quality control filtering, a total of 107,092 sequences were deemed suitable for taxonomic assignment, averaging 2,677 sequence reads per sample. Sequence reads were clustered as 2,221 OTUs and 43 different bacterial phyla (**Figure 2**, Supplementary Table S1). Phyla that represented more than 10% of the sequenced community at any one sampling depth included Actinobacteria, Atribacteria, Bacteroidetes, Planctomycetes, and the sub-phyla Alpha- and Gammaproteobacteria. Cyanobacteria sequences consisted of chloroplast 16S rRNA genes, suspected to be fossilized DNA or cells, derived from surface waters and well preserved in this anoxic environment. Other phyla that represented more than 1% of the sequenced community included Acidobacteria, Chloroflexi, Firmicutes, Spirochaetes, sub-phylum Deltaproteobacteria, Aminicenantes (formerly Candidate Division OP8), Latescibacteria (formerly Candidate Division WS3), and Candidate DivisionWS1. Similar to Antarctic sediments of the Ross Sea (Carr et al., 2013), it appears that the bacterial communities were dominated by heterotrophic organisms, based on the energy metabolisms of closely related cultured organisms. For example the majority of Bacteroidetes OTUs were classified as Sphingobacteria, anaerobic heterotrophs often associated with hydrolytic and fermenting abilities (Cottrell and Kirchman, 2000). Additionally, most of the sequenced Actinobacteria OTUs were classified as Acidimicrobineae, a group capable of breaking down complex organic material (McCarthy and Williams, 1992; Schrempf, 2001; Vorob’ev et al., 2007).

**FIGURE 3:**
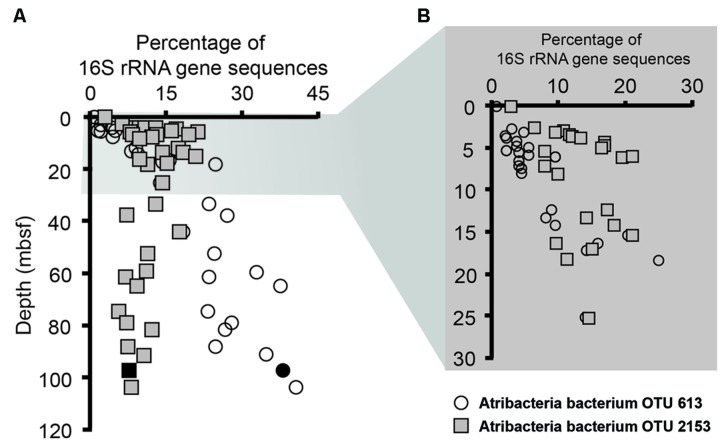
**Relative abundances of Atribacteria OTUs 2153 (squares) and 613 (circles) sequences determined by pyrosequencing. (A)** This includes samples throughout the entire core, while **(B)** focuses on the top 30 m below seafloor (mbsf). Solid black symbols represent the sample utilized for single cell genomics.

**FIGURE 4:**
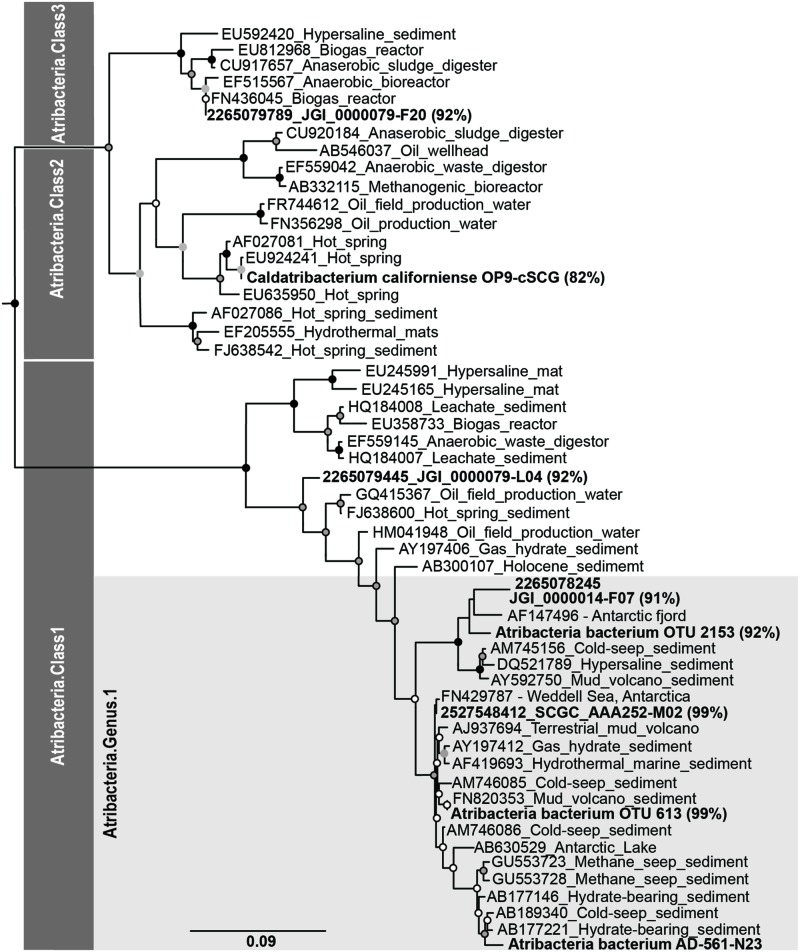
**Phylogenetic associations of Atribacteria based on the 16S rRNA gene.** Bold sequences indicate sequences from this study, while sequences from other studies are indicated with Genbank accession ID numbers. Percentages represent the 16S rRNA gene similarity to the subsurface Antarctic bacterium SCGC AD-561-N23 single amplified genome according to BLAST analyses.

Notably, sequences related to Atribacteria increased in relative abundance with depth in the sediment, ranging from less than 5% in shallow samples (0.05 mbsf) and became the dominant group in deeper samples, representing over 50% at 103.6 mbsf (**Figure 2**). The vast majority (94%) of the Atribacteria sequences grouped within two OTUs, referred to as OTU 613 and OTU 2153. OTU 2153 dominated in sediment depths above the maximum methane concentration (12.8 mM), while OTU 613 dominated in the deeper sediment depths (**Figure 3**). Phylogenetic analysis of these two OTUs revealed that they cluster most closely to sequences from other anoxic marine sediment samples as opposed to sequences from bioreactors or terrestrial hot springs (**Figure 4**). Moreover, OTU 613 grouped closely (99–100% sequence similarity, Supplementary Table S2) to sequences from other Antarctica lake sediment (Bowman et al., 2000), suggesting that geographic location as well as environmental conditions contribute to phylogenetic diversity. Overall, the microbial composition and relative sequence abundances of Atribacteria measured at this site were similar to those observed within methane-rich hydrate-bearing sediments (Inagaki et al., 2003, 2006; Newberry et al., 2004). The lack of a bottom simulating reflector in this basin (Escutia et al., 2008) and constant salinity values suggest the absence of hydrates in the Adélie Basin; however, methane concentrations at this site are still rather high (**Figure 2**), supporting an association of Atribacteria with high-methane conditions.

### Genome Characteristics of a Single Atribacteria Cell from Deep Adélie Basin Sediment

Sediments for the Adélie Basin were frozen upon collection, and thus considered to be non-ideal for single cell techniques. However, given our desire to focus on the functionality of Atribacteria and the relatively large proportion of Atribacteria sequence reads, we attempted the challenge of cell sorting. Flow cytometric scattergrams of diluted sediment slurries indicated probable success for sorting intact cells (Supplementary Figure S2). A total of 768 potential cells were sorted. MDA reactions resulted in only eight successful amplifications, of which only one was identified as Atribacteria. Considering that these samples had already experienced several freeze thaw cycles, we hypothesize that cell resistance to lysis techniques is likely responsible for these limited results. However, the successful isolation of an Atribacteria cell does indicate the potential use of single cell techniques with frozen sediments, especially if more appropriate cell lysis procedures can be adapted. Given the amount of existing frozen sediment samples that have been collected for biological studies throughout the history of oceanic drilling programs, improved techniques could unlock a huge amount of currently stored genomic information.

The one successful single cell amplified genome (Atribacteria bacterium SCGC AD-561-N23) contained a 16S rRNA gene sequence that shared 99% sequence similarity to the Atribacteria OTU 613 (**Figure 4**), which was the dominant OTU in the deeper samples (**Figure 3**). To our knowledge, this Atribacteria cell is the first reported cell to be isolated from a deep (>10 mbsf) subsurface sediment. While the single cell DNA amplification success rate was lower than those from other sedimentary environments (Lloyd et al., 2013; Kaster et al., 2014; Wasmund et al., 2014), genome amplification of any cell is remarkable considering that the samples were not preserved for single cell sorting and amplification upon collection. The final genome assembly size was 209 kbp with 24 scaffolds (**Table 2**). A total of 207 coding sequences were annotated. Coding sequences identified as putative proteins were most abundant (70 coding sequences), followed by those related to replication, transcription, and translation functions (Supplementary Table S3). Based on the identification of 21 out of 139 conserved bacterial genes (Rinke et al., 2013), genome completeness was estimated to be 15%. Amplification bias during MDA reactions is the suspected cause of the low genomic coverage and is not uncommon for environmental samples (Raghunathan et al., 2005; Lasken, 2007).

**Table 2:** Genomic characteristics of Atribacteria bacterium SCGC AD-561-N23 from deep Antarctica sediment.

Characteristic	Value
Assembly size	209,267 bp
Contigs	24
Longest contig	27,948 bp
N50	14516
Coding sequences	207
Non-hypothetical proteins	137
Ribosomal proteins	12
GC content	38%
Level of completeness	15%

Phylogenic reconstruction of the candidate phylum Atribacteria based on the 16S rRNA gene identifies three different classes (Yarza et al., 2014). According to these taxonomic classifications, Atribacteria bacterium SCGC AD-561-N23, OTU 613, and OTU 2153 are all affiliated with candidate taxonomic unit (CTU) Atribacteria.Class1, and more specifically Atribacteria.Genus1 (**Figure 4**). A comparison between the SAG of this study and SAGs sampled from terrestrial and aquatic environments revealed a range of sequence similarity (82–98%), with higher similarity to SAGs from cool, saline environments than to SAGs from anaerobic bioreactors and terrestrial hot springs (**Figure 4**). Atribacteria bacterium SCGC AAA252-M02 and Atribacteria bacterium JGI 0000014-F07 share the highest percentage of similarity with the SAG from this study (98 and 91%, respectively), with the former grouping in the same candidate genus (**Figure 4**). Atribacteria bacterium JGI 0000079-L04 is also identified as Atribacteria.Class1 but falls outside of the Atribacteria.Genus1 classification. Atribacteria bacterium JGI 0000079-F20 and *Ca. C. californiense* are affiliated with Atribacteria.Class3 and Atribacteria.Class2, respectively.

Similar grouping trends are observed when comparing GC content and ANI of the SAGs. The GC content for the partial genome of Atribacteria bacterium SCGC AD-561-N23 was 38%, which is similar to most other Atribacteria SAGs (35–37%, **Table 1**), although *C. californiense* with a hot-spring origin has a GC content of 55% (Dodsworth et al., 2013). The calculated ANI values of shared oligonucleotide sequences illustrate that the Atribacteria bacterium SCGC AD-561-N23 SAG is 87 and 77% similar to SAGs collected from brackish water and lagoon sediments, respectively, while it is only 59% similar to the *C. californiense* from a hot spring (**Table 1**). Genome coverage for ANI comparisons against *C. Californiense*, Atribacteria bacterium JGI 0000079-F20 and Atribacteria bacterium JGI 0000014-F07 were very low (<4% genome coverage) and should be interpreted which caution (Richter and Rosselló-Móra, 2009). Nevertheless, collectively, these similarity trends suggest that the SAG of this study is most comparable to organisms collected from marine-like settings; therefore these SAGs are expected to share physiological traits.

### Heterotrophic Metabolic Potential of Sediment Atribacteria

**FIGURE 5:**
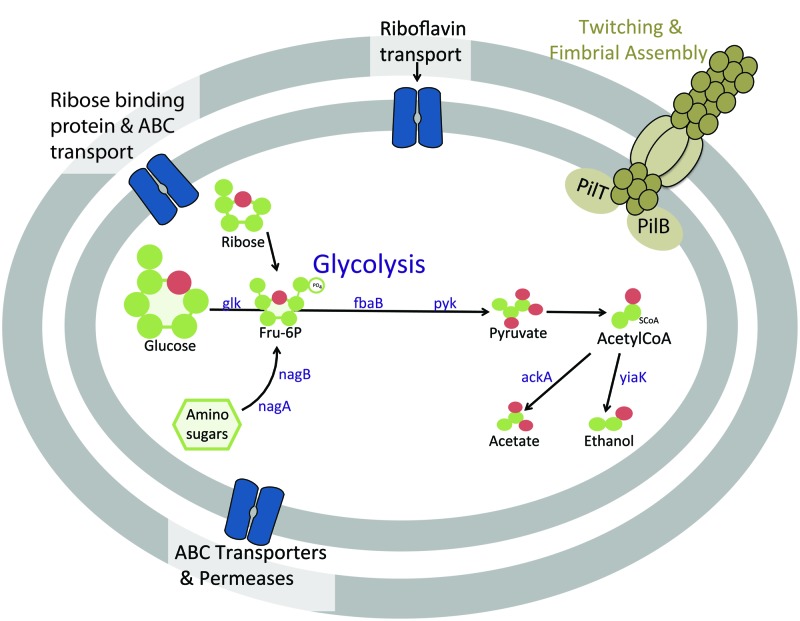
**Cartoon schematic of proteins in Atribacteria bacterium SCGC AD-561-N23 associated with heterotrophic metabolism and motility functions.** Abbreviations: ABC, ATP-binding cassette; ackA, acetate kinase; glk, glucose/hexose kinase; fbaB, fructose 1-6 bisphosphate aldolase; nagA, *N*-aceylglucosamine-6-phosphate amidohydrolase; nagB, glucosamine-6-phosphate deaminase; pyk, pyruvate kinase; yaiK, alcohol dehydrogenase.

Similar to previously investigated Atribacteria SAGs, the subsurface Atribacteria bacterium SCGC AD-561-N23 appears to be heterotrophic, based on genomic observation of sugar and amino-sugar metabolic and fermentation pathways (**Figure 5**). Sugar metabolism by Atribacteria bacterium SCGC AD-561-N23 is evident by several sugar membrane transporters and permeases, including those specifically for ribose sugar (**Figure 5**, Supplementary Table S4, IMG ID 2590808876). Coding sequences for hexose kinase (IMG ID 2590809017, EC:2.7.1.2), fructose 1-6 bisphosphate aldolase (IMG ID 2590809081 EC:4.1.2.13), and pyruvate kinase genes (IMG ID 2590808886, EC:2.7.1.40) indicate Embden-Meyerhof glycolysis and the anaerobic catabolism of six carbon sugars.

When considering the potential for fermentation by sediment Atribacteria (**Figure 5**, Supplementary Table S3), the reduction of pyruvate to CO_2_ and acetyl-CoA is evident by coding sequences for pyruvate:ferredoxin oxidoreductase (IMG IDs 2590808972 and 2590808975) and the required co-factor thiamine pyrophosphate (IMG ID 2590808973; Uyeda and Rabinowitz, 1971). The presence of acetate kinase (2590808976, EC: 2.7.2.1) and alcohol dehydrogenase (2590809027, EC:1.1.1.1) suggests additional fermentation of pyruvate to acetate or ethanol may be possible. The same genes were identified within *C. californiense*, and the more closely related Atribacteria SAGs SCGC AAA252-M02, further supporting the possibility of fermentation pathways in Atribacteria bacterium SCGC AD-561-N23.

Amino-sugar metabolism is also evident in Atribacteria bacterium SCGC AD-561-N23 (**Figure 5**). Amino sugars such as *N*-acetyl-D-glucosamine-6-phopate could be transported into the cell by ATP-driven ABC transporters encoded within this SAG (IMG ID 2590809113). Once in the cell, the amino sugars could be converted to fructose-6-phosphate by *N*-aceylglucosamine-6-phosphate amidohydrolase (IMG IDs 2590808895 and 2590809033, EC:3.5.1.22) and glucosamine-6-phosphate deaminase (2590809032, EC:3.5.99.6). The resulting fructose-6-phosphate may be further degraded via glycolysis as noted above. Additional acetate and NH_3_ products could be potentially used in many processes, including amino acid metabolism, lipid synthesis, or ethanol fermentation.

### Metabolic and Functional Diversity within the Atribacteria Phylum

There is no evidence that Atribacteria are directly involved in methane production or consumption, although this might be speculated based on the co-occurrence of Atribacteria in methane rich sediment (**Figure 3**). Instead, the partial genome of Atribacteria bacterium SCGC AD-561-N23 suggests that Atribacteria produce fermentation products such as acetate, ethanol and CO_2_ that could be used as substrates by methanogenic organisms. Few archaea, including methanogens, were observed in the pyrosequencing survey (less than <0.2% total sequences; Carr, 2012). This absence may be the result of primer bias (Teske and Sørensen, 2007), however, we hypothesize that methane-producing organisms are in relatively low relative abundance, as has been observed in other methane-rich sediment environments (Inagaki et al., 2006; Colwell et al., 2008). Active methanogenesis is evident from increasing methane concentrations and an enrichment of dissolved inorganic carbon ^13^C values with sediment depth (Carr, 2012). A similar relationship between heterotrophic Atribacteria and methanogens has been proposed within the water column of a meromictic, brackish lake (Gies et al., 2014). From that environment, the genome of Atribacteria bacterium SCGC AAA252-M02 indicates acetate oxidation and the production of CO_2_ and H_2_ using the Wood–Ljungdahl pathway in reverse (Müller et al., 2013). Atribacteria bacterium JGI 0000079-L04, which is classified within the same class as Atribacteria bacteria SCGC AAA252-M02 and SCGC AD-5601-N23, also contains many coding sequences for the Wood–Ljungdahl pathway (Nobu et al., 2015). Atribacteria bacterium SCGC AD-5601-N23 lacks evidence of this pathway, but given the low genome coverage, and the close relation to SCGC AAA252-M02 and JGI 0000079-L04, it is possible that bacterium SCGC AD-5601-N23 also contains these genes. However, regardless if Atribacteria bacterium SCGC AD-5601-N23 is capable of both acetate oxidation and sugar fermentation, or sugar fermentation alone, the interpretation here presents preliminary evidence for a heterotrophic lifestyle and the production of simple carbon substrates that could fuel methanogenesis. Given the importance of fermenters to methanogenic communities (Kirchman et al., 2014), our initiatory results provide compelling motivation for future investigations of sedimentary Atribacteria. We expect that these additional studies will provide valuable insight to the heterotrophic capabilities of Atribacteria, their potential indirect interactions with methanogens and ultimately, the biogeochemistry of carbon cycling and methane production in marine sediment.

In summary, pyrosequencing of the 16S rRNA gene revealed a large relative abundance of sequence reads classified as Atribacteria within sediments of the Adélie Basin. While samples were not originally collected and preserved for single cell analyses, we demonstrated the first successful isolation of a deep (>10) subsurface cell. This success highlights the potential application of single cell techniques to stored frozen sediment samples, but also recognizes the potential need for more aggressive cellular lysis procedures. The preliminary genomic data presented here, along with sediment biogeochemistry and molecular biology suggests that the abundant Atribacteria in deep methane-rich marine sediment are heterotrophic, providing fermentative precursors for methane production by other organisms. Although there is no known cultured representative from the Atribacteria phylum, culture-independent single cell genomes derived from terrestrial and aquatic environments suggest that Atribacteria are similarly heterotrophic (Webster et al., 2006a; Dodsworth et al., 2013; Rinke et al., 2013). The sediment Atribacteria bacterium SCGC AD-561-N23 SAG provides ample evidence for sugar and amino-sugar metabolism producing fermentation products such as acetate, ethanol, and CO_2_. Thus, Atribacteria are hypothesized to play a key role in regulating both the production of methane in marine sediment and the diversity of methane producers, depending on the abundance and type of fermentation products. Moreover, this study confirms that high latitude marine sediment from an organic-rich and highly productive continental margin environment displays similar microbial diversity patterns to lower latitude deep sediment, suggesting that attempts to constrain global sediment diversity (D’Hondt et al., 2004; Carr et al., 2013) can be expanded to include higher latitude sites with limited available data.

## Conflict of Interest Statement

The authors declare that the research was conducted in the absence of any commercial or financial relationships that could be construed as a potential conflict of interest.
