# Supplementary material for: Abundant Atribacteria in deep marine sediment from the Adélie Basin, Antarctica
Source: Front Microbiol. 2015 Aug 26;6:872. doi: 10.3389/fmicb.2015.00872 (PMC4549626; doi:10.3389/fmicb.2015.00872)
Supplement: Supplementary file 1 [file Data_Sheet_1.DOCX]

Supplemental Materials for "Abundant Atribacteria in deep marine sediment from the Adélie Basin, Antarctica **"**

Authors:

Stephanie A. Carr^1*^, Beth N. Orcutt^2^, Kevin W. Mandernack^3^, John R. Spear^1^

1: Colorado School of Mines, Department of Civil and Environmental Engineering, Golden, CO, 80401 USA

2: Bigelow Laboratory for Ocean Sciences, East Boothbay, ME, 04544 USA

3: Indiana University – Purdue University Indianapolis, Department of Earth Sciences, Indianapolis, Indiana, 46202 USA

*Correspondence:
Stephanie Carr
Colorado School of Mines
Department of Civil and Environmental Engineering
1500 Illinois Street
Golden, CO, 80401, USA
[scarr@mymail.mines.edu](mailto:scarr@mymail.mines.edu)

**Selected Fasta Files**

>Atribacteria_bacterium_SCGC_AD-561_N23

CCTAACACATGCAAGTCGAACGAGAAGCTAACTTCTGATTCCTTCGGGATGATGAAGTTGGTAGACAGTGGCGAACGGGTGAGTAACGCGTGGGTAATCTACCCTGTAAGTGGGGGATAACCCTTCGAAAGGAGGGCTAATACCGCATAATATCTTTACTTCTCAAGAAGCAAAGATTAAAGATGGCCTCTATACTATGCTATCACTTCAGGATGAGCCCGCGTCCTATTAGTTAGTTGGTGGGGTAATGGCCTACCAAGACCACAATGGGTAGCCGGTCTGAGAGGATGTACGGCCACACTGGGACTGAGATACGGCCCAGACTCCTACGGGAGGCAGCAGTGGGGAATATTGCGCAATGGGGGAAACCCTGACGCAGCGACGCCGCGTGGATGATGAAGGCCTTTGGGTTGTAAAATCCTGTTTTGGGGGAAGAAAGCTTAAGAGTTCAATAAACCCTTAAGCCTGACGGTACCCCAAGAGAAAGCTCCGGCTAATTATGTGCCAGCAGCCGCGGTAATACATAAGGAGCAAGCGTTATCCGGAATTATTGGGCGTAAAGAGCTCGTAGGCGGTCTTAAAAGTCAGGTGTGAAATTATCAGGCTCAACCTGATAAGGTCATCTGAAACTTTAAGACTTGAGGTTAGAAGAGGAAAGTGGAATTCCCGGTGTAGCGGTGAAATGCGTAGATATCGGGAGGAACACCAGTGGCGAAGGCGGCTTTCTGGTCTATCTCTGACGCTGAGGAGCGAAAGCTAGGGGAGCAAACGGGATTAGATACCCCGGTAGTCCTAGCTGTAAACGATGGATACTAGGTGTGGGAGGTATCGACCCCTTCTGTGCCGTAGCTAACGCATTAAGTATCCCGCC

>Atribacteria_OTU_613

TACATAAGGAGCAAGCGTTATCCGGAATTATTGGGCGTAAAGAGCTCGTAGGCGGTCTTAAAAGTCAGGTGTGAAATTATCAGGCTCAACCTGATAAGGTCATCTGAAACTTTAAGACTTGAGGATAGAAGAGGAAAGTGGAATTCCCGGTGTAGCGGTGAAATGCGTAGATATCGGGAGGAACACCAGTGGCGAAGGCGGCTTTCTGGTCTATCTCTGACGCTGAGGAGCGAAAGCTAGGGGAGCAAACGGGATTAGATACCCCGGTAGTCCTAGCTGTAAACGATGGATACTAGGTGTGGGAGGTATCGACCCCTTCTGTGCCGTAGCTAACGCATTAAGTATCCCGCCTGGGGAGTACGGTCGCAAGGCT

>Atribacteria_OTU_2153

TACATAAGGAGCAAGCGTTGTCCGGAATTATTGGGCGTAAAGGGCTCGTAGGCGGTTTTAAAAGTCAGATGTTAAATTACCAGGCTTAACCTGGTGCTGTCATCTGAAACTTTAAGACTTGAGGTCAGAAGAGGAAAGTGGAATTCCCGGTGTAGCGGTGAAATGCGTAGATATCGGGAGGAACACCAGTGGCGAAGGCGGCTTTCTGGTCTGACTCTGACGCTGAGGAGCGAAAGCTAGGGGAGCAAACAGGATTAGATACCCTGGTAGTCCTAGCTGTAAACGATGGATACTAGGTGTGGGAGGTATCGACCCCTTCTGTGCCGCAGCTAACGCATTAAGTATCCCGCCTGGGGAGTACGGTCGCAAGGCT

**Supplemental Figure 1.** Relative abundances of 16S rRNA gene sequences from bacterial phyla and subphyla detected from extraction replicates. Pearson correlation coefficients between replicate samples are greater than 0.96.

**
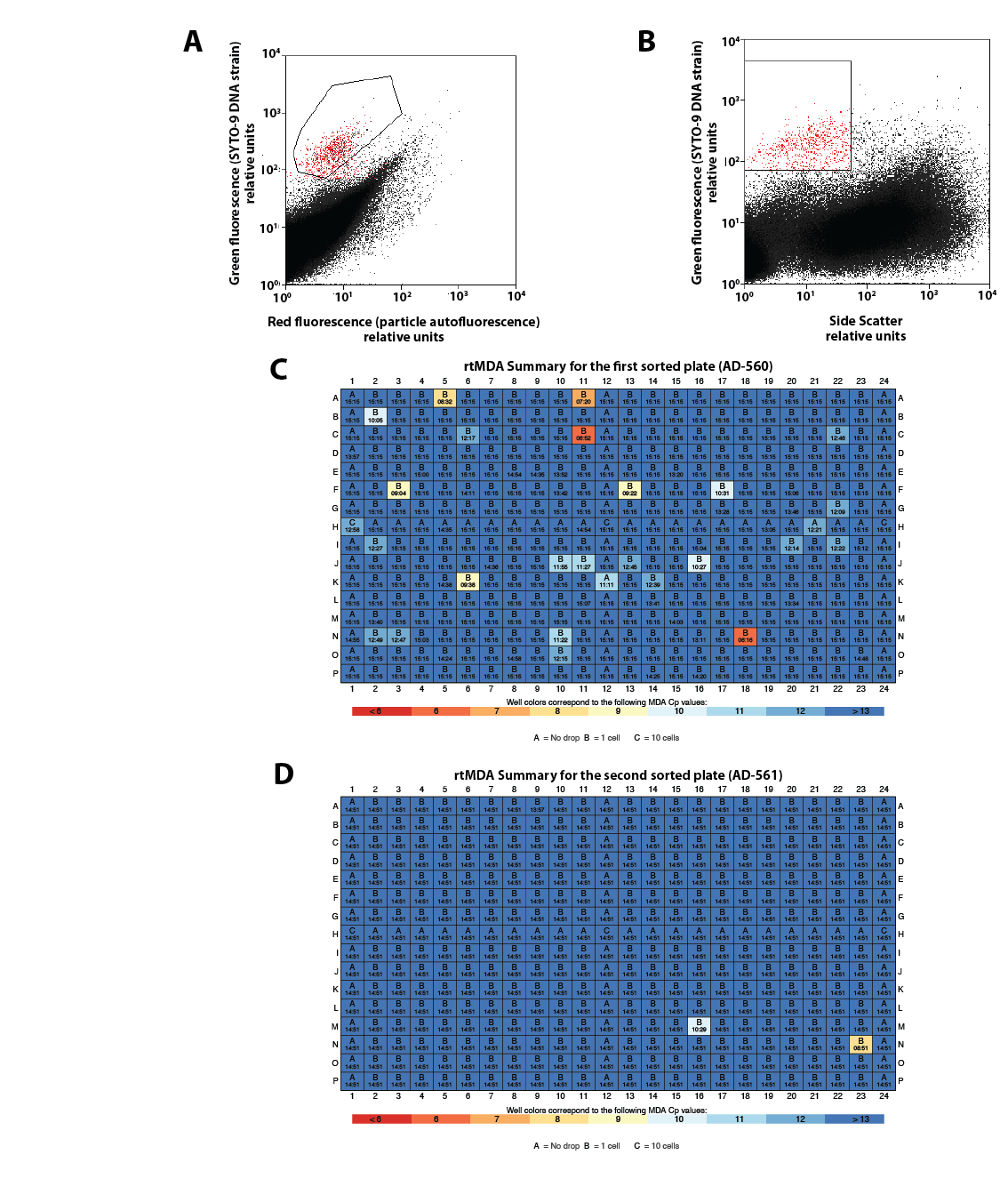
**

**Supplemental Figure 2**: (A,B) Flow cytometric scattergrams of a sediment slurry collected from the Adélie Basin, Antarctica. Red regions identify particles that suspected to be intact cells. MDA results for sorted cells (C,D). Both plates were subjected to physical lysis treatments, and the second plate (D) also experienced an alkaline lysis treatment. The Atribacteria cell utilized for single cell genomics originated from the second plate, well N23.

**Supplemental Table 1**. OP9 species with high similarity to OTU 613 (>98%) BLASTn search against the NCBI database ([Altschul et al., 1990](#_ENREF_1)).

| Accession Number | Organism Name | Length (bp) | Similarity to OTU | Site description | Reference |
| --- | --- | --- | --- | --- | --- |
| OTU 613 | | | | | |
| AF142824 | uncultured bacterium BURTON-4 | 1005 | 99% | sediments from Burton Lake, Antarctica | Bowman et al, 2000 |
| AF147496 | uncultured bacterium SCALE-16 | 526 | 99% | anoxic sediments from Scale Lake, Antarctica | Bowman et al, 2000 |
| AF142787 | uncultured bacterium ACE-5 | 1006 | 99% | anoxic sediments from Ace Lake, Antarctica | Bowman et al, 2000 |
| AF142966 | uncultured bacterium TAYNAYA-18 | 1010 | 99% | anoix sediments fromTaynaya Bay, Antarctica | Bowman et al, 2000 |
| AF142820 | uncultured bacterium ACE-45 | 1010 | 100% | anoxic sediments from Ace Lake, Antarctica | Bowman et al, 2000 |
| AB630529 | Uncultured bacterium Clone MPB1-147 | 1510 | 99% | Aquatic moss pillars Hotoke-Ike Lake | ([Nakai et al., 2012](#_ENREF_2)) |

**Supplemental Table 2**. Relative abundances of bacterial phyla for all sample depths

| Depth | Acidobacteria | Actinobacteria | Bacteroidetes | Aminicenantes | Atribacteria | Ca. division WS1 |
| --- | --- | --- | --- | --- | --- | --- |
| 0.05 | 2.8 | 6.8 | 24.7 | 0.6 | 4.0 | 0.3 |
| 2.75 | 1.5 | 8.3 | 25.5 | 0.8 | 10.3 | 0.5 |
| 2.95 | 1.4 | 5.5 | 19.8 | 1.6 | 15.8 | 1.2 |
| 3.15 | 1.0 | 7.0 | 20.5 | 2.9 | 16.0 | 0.9 |
| 3.45 | 2.1 | 5.5 | 22.2 | 1.8 | 14.3 | 0.4 |
| 3.65 | 1.3 | 5.4 | 18.4 | 1.3 | 15.2 | 0.5 |
| 3.85 | 0.7 | 2.9 | 15.4 | 1.1 | 17.0 | 0.4 |
| 4.45 | 0.4 | 1.7 | 14.2 | 1.0 | 21.8 | 1.0 |
| 4.75 | 2.1 | 7.1 | 14.1 | 1.6 | 21.7 | 0.9 |
| 5.05 | 1.7 | 3.6 | 16.2 | 1.7 | 22.7 | 0.9 |
| 5.35 | 1.4 | 3.9 | 10.5 | 1.1 | 10.8 | 0.4 |
| 5.65 | 1.7 | 5.0 | 18.9 | 1.5 | 18.7 | 0.7 |
| 5.95 | 1.8 | 7.9 | 12.5 | 3.3 | 29.2 | 1.8 |
| 6.15 | 1.0 | 5.4 | 13.8 | 2.4 | 29.8 | 0.7 |
| 6.65 | 0.8 | 3.6 | 13.6 | 2.0 | 18.6 | 1.1 |
| 7.15 | 1.3 | 7.9 | 21.0 | 1.9 | 12.9 | 0.6 |
| 7.65 | 0.9 | 3.8 | 11.0 | 1.9 | 17.7 | 1.1 |
| 8.15 | 1.0 | 3.1 | 13.4 | 2.0 | 15.1 | 0.5 |
| 12.35 | 2.1 | 5.0 | 11.0 | 3.1 | 28.3 | 1.6 |
| 13.35 | 1.3 | 4.1 | 22.6 | 3.6 | 23.8 | 1.1 |
| 14.25 | 0.8 | 4.0 | 13.2 | 3.3 | 29.5 | 0.7 |
| 15.35 | 0.9 | 5.1 | 5.4 | 4.5 | 43.2 | 3.8 |
| 16.35 | 1.0 | 3.4 | 8.2 | 2.9 | 28.4 | 1.5 |
| 17.26 | 0.5 | 4.8 | 5.6 | 5.5 | 30.8 | 2.9 |
| 18.37 | 1.9 | 4.1 | 7.3 | 4.8 | 39.4 | 2.8 |
| 25.24 | 1.0 | 3.6 | 11.6 | 3.1 | 29.4 | 2.0 |
| 33.23 | 1.9 | 4.2 | 3.4 | 4.2 | 39.3 | 3.2 |
| 37.67 | 1.7 | 6.6 | 2.4 | 4.0 | 36.0 | 4.9 |
| 44.39 | 1.1 | 6.0 | 2.8 | 1.9 | 37.0 | 2.6 |
| 52.51 | 0.3 | 1.5 | 5.0 | 2.0 | 37.5 | 4.5 |
| 59.41 | 0.5 | 2.2 | 1.2 | 2.2 | 49.0 | 6.4 |
| 61.79 | 0.8 | 4.5 | 2.3 | 2.6 | 32.3 | 3.9 |
| 64.76 | 1.8 | 5.8 | 2.7 | 3.3 | 48.4 | 4.1 |
| 74.47 | 2.0 | 6.8 | 2.3 | 2.6 | 29.7 | 3.4 |
| 78.78 | 1.0 | 4.6 | 2.4 | 2.9 | 36.6 | 7.5 |
| 81.56 | 1.7 | 7.0 | 3.1 | 2.2 | 39.6 | 5.0 |
| 88.29 | 1.5 | 6.0 | 2.6 | 2.7 | 34.1 | 5.3 |
| 91.29 | 0.9 | 4.4 | 3.6 | 4.3 | 47.3 | 5.1 |
| 97.41 | 0.7 | 3.5 | 2.4 | 4.9 | 49.2 | 6.1 |
| 103.64 | 0.7 | 1.7 | 2.2 | 4.2 | 51.2 | 4.9 |

**Supplemental Table 2 (continued)**. Relative abundances of bacterial phyla for all sample depths

| Depth | Latescibacteria | Chloroflexi | Cyanobacteria | Firmicutes | Gemmatimonadetes | Planctomycetes |
| --- | --- | --- | --- | --- | --- | --- |
| 0.05 | 2.7 | 2.6 | 1.7 | 1.7 | 1.1 | 9.9 |
| 2.75 | 3.3 | 2.9 | 1.3 | 2.4 | 0.6 | 11.1 |
| 2.95 | 1.7 | 3.7 | 0.2 | 3.0 | 0.9 | 9.6 |
| 3.15 | 1.5 | 1.5 | 0.8 | 4.6 | 0.8 | 10.1 |
| 3.45 | 3.5 | 3.9 | 2.6 | 1.9 | 0.7 | 8.5 |
| 3.65 | 3.0 | 2.9 | 10.1 | 4.2 | 0.3 | 10.7 |
| 3.85 | 2.0 | 1.2 | 22.1 | 8.7 | 0.3 | 5.3 |
| 4.45 | 1.9 | 1.8 | 3.2 | 9.3 | 0.3 | 8.2 |
| 4.75 | 1.3 | 1.8 | 9.3 | 4.6 | 0.4 | 10.4 |
| 5.05 | 1.8 | 1.8 | 3.5 | 4.4 | 0.7 | 8.6 |
| 5.35 | 0.9 | 1.6 | 28.7 | 3.8 | 0.2 | 5.1 |
| 5.65 | 1.0 | 2.5 | 12.4 | 2.5 | 0.9 | 8.0 |
| 5.95 | 0.9 | 3.0 | 0.4 | 8.2 | 0.7 | 7.7 |
| 6.15 | 2.2 | 2.0 | 4.2 | 5.7 | 0.2 | 8.6 |
| 6.65 | 2.0 | 2.1 | 24.4 | 6.0 | 0.3 | 5.3 |
| 7.15 | 2.1 | 3.3 | 4.6 | 3.4 | 0.5 | 8.6 |
| 7.65 | 1.0 | 2.4 | 25.5 | 5.3 | 0.3 | 6.8 |
| 8.15 | 1.2 | 2.0 | 35.2 | 3.2 | 0.4 | 5.0 |
| 12.35 | 1.1 | 3.1 | 0.4 | 3.1 | 0.4 | 10.5 |
| 13.35 | 0.9 | 2.9 | 0.3 | 5.7 | 0.8 | 7.7 |
| 14.25 | 1.1 | 3.4 | 0.1 | 5.0 | 0.1 | 8.4 |
| 15.35 | 0.3 | 4.7 | 0.1 | 5.2 | 0.2 | 5.8 |
| 16.35 | 0.4 | 3.5 | 0.3 | 5.3 | 0.5 | 8.8 |
| 17.26 | 0.5 | 3.2 | 0.5 | 11.4 | 0.2 | 7.1 |
| 18.37 | 0.6 | 3.4 | 0.2 | 3.5 | 0.3 | 8.9 |
| 25.24 | 0.2 | 2.6 | 0.9 | 6.8 | 0.2 | 6.7 |
| 33.23 | 0.5 | 2.2 | 0.4 | 3.2 | 0.4 | 9.0 |
| 37.67 | 0.3 | 3.1 | 0.5 | 5.8 | 0.1 | 8.9 |
| 44.39 | 0.2 | 2.4 | 0.2 | 7.9 | 0.2 | 6.4 |
| 52.51 | 0.2 | 4.0 | 0.3 | 10.3 | 0.1 | 7.2 |
| 59.41 | 0.5 | 3.8 | 0.0 | 8.1 | 0.0 | 4.9 |
| 61.79 | 0.1 | 3.4 | 1.7 | 3.7 | 0.0 | 10.0 |
| 64.76 | 0.2 | 3.5 | 0.0 | 2.1 | 0.1 | 4.9 |
| 74.47 | 0.1 | 2.8 | 0.2 | 8.1 | 0.1 | 5.6 |
| 78.78 | 0.1 | 3.7 | 0.2 | 5.9 | 0.1 | 6.3 |
| 81.56 | 0.3 | 3.6 | 0.4 | 5.2 | 0.1 | 5.8 |
| 88.29 | 0.2 | 2.6 | 0.0 | 6.1 | 0.1 | 8.6 |
| 91.29 | 0.2 | 2.7 | 0.1 | 4.0 | 0.0 | 5.1 |
| 97.41 | 0.4 | 2.5 | 0.1 | 3.4 | 0.2 | 6.5 |
| 103.64 | 0.1 | 2.3 | 0.2 | 6.6 | 0.1 | 4.8 |

**Supplemental Table 2 (continued)**. Relative abundances of bacterial phyla for all sample depths

| Depth | Spirochaetes | Proteobacteria | Proteobacteria | Proteobacteria | Other | Archaea | Total Seq. |
| --- | --- | --- | --- | --- | --- | --- | --- |
| 0.05 | 2.7 | 9.3 | 5.9 | 17.4 | 3.9 | 1.5 | 2483 |
| 2.75 | 2.1 | 10.1 | 4.5 | 10.2 | 2.7 | 1.4 | 2518 |
| 2.95 | 3.2 | 6.8 | 3.7 | 16.5 | 4.0 | 1.4 | 3758 |
| 3.15 | 3.6 | 5.4 | 3.4 | 14.5 | 5.0 | 0.4 | 3873 |
| 3.45 | 2.5 | 5.7 | 4.4 | 13.8 | 3.9 | 2.2 | 3022 |
| 3.65 | 4.0 | 6.2 | 3.8 | 7.9 | 3.3 | 1.3 | 2982 |
| 3.85 | 3.0 | 3.7 | 2.6 | 9.1 | 3.4 | 0.6 | 2839 |
| 4.45 | 4.1 | 3.4 | 5.3 | 14.8 | 5.0 | 2.2 | 3345 |
| 4.75 | 2.1 | 6.1 | 5.7 | 6.7 | 2.9 | 1.4 | 2571 |
| 5.05 | 2.4 | 9.8 | 4.8 | 10.2 | 3.3 | 1.7 | 3646 |
| 5.35 | 3.1 | 4.9 | 3.8 | 16.3 | 2.1 | 1.2 | 2663 |
| 5.65 | 1.6 | 6.3 | 4.6 | 8.8 | 3.2 | 1.4 | 2515 |
| 5.95 | 2.6 | 6.4 | 2.8 | 5.5 | 3.4 | 0.6 | 2436 |
| 6.15 | 3.1 | 4.6 | 3.9 | 7.3 | 3.0 | 1.7 | 2994 |
| 6.65 | 2.6 | 4.0 | 3.2 | 4.5 | 4.0 | 1.9 | 2871 |
| 7.15 | 2.9 | 8.5 | 3.3 | 11.1 | 4.5 | 1.3 | 2818 |
| 7.65 | 2.5 | 6.3 | 3.2 | 5.6 | 2.8 | 1.7 | 3056 |
| 8.15 | 1.5 | 5.6 | 2.0 | 4.2 | 3.3 | 1.2 | 2505 |
| 12.35 | 3.1 | 10.4 | 3.2 | 8.2 | 3.4 | 1.9 | 2970 |
| 13.35 | 2.9 | 5.3 | 3.3 | 8.1 | 3.2 | 2.1 | 3257 |
| 14.25 | 3.4 | 6.8 | 3.7 | 10.4 | 3.2 | 2.7 | 3313 |
| 15.35 | 2.2 | 4.1 | 2.6 | 6.5 | 3.4 | 1.8 | 2650 |
| 16.35 | 2.0 | 6.1 | 3.4 | 16.0 | 5.0 | 3.3 | 2732 |
| 17.26 | 2.2 | 6.5 | 2.4 | 9.3 | 5.0 | 1.3 | 2708 |
| 18.37 | 1.4 | 8.1 | 2.2 | 5.9 | 3.1 | 2.0 | 3246 |
| 25.24 | 1.6 | 6.3 | 3.9 | 13.3 | 3.9 | 2.5 | 2505 |
| 33.23 | 1.2 | 8.4 | 2.1 | 10.1 | 3.9 | 2.3 | 2333 |
| 37.67 | 1.0 | 7.7 | 2.3 | 5.5 | 5.4 | 3.6 | 1905 |
| 44.39 | 1.5 | 12.5 | 2.4 | 7.5 | 4.8 | 2.3 | 1712 |
| 52.51 | 1.5 | 2.6 | 2.3 | 12.2 | 3.8 | 3.8 | 1325 |
| 59.41 | 1.1 | 4.3 | 1.8 | 4.9 | 3.9 | 3.7 | 2452 |
| 61.79 | 0.7 | 10.8 | 1.6 | 13.9 | 4.2 | 3.4 | 2097 |
| 64.76 | 0.7 | 5.4 | 2.3 | 6.1 | 3.9 | 4.9 | 2639 |
| 74.47 | 1.3 | 5.7 | 1.7 | 20.3 | 2.4 | 4.9 | 1930 |
| 78.78 | 1.9 | 2.2 | 1.8 | 15.2 | 3.4 | 4.3 | 1971 |
| 81.56 | 0.9 | 6.1 | 1.7 | 8.0 | 5.2 | 4.1 | 2233 |
| 88.29 | 2.1 | 2.5 | 1.9 | 14.4 | 4.8 | 4.6 | 2285 |
| 91.29 | 2.7 | 4.9 | 1.7 | 2.9 | 2.9 | 7.4 | 2138 |
| 97.41 | 1.0 | 3.0 | 1.1 | 8.6 | 3.5 | 3.0 | 3025 |
| 103.64 | 3.5 | 2.1 | 1.3 | 8.8 | 3.4 | 1.7 | 2771 |

**Supplemental Table 3**. Genes of various metabolic functions in the partial genome of Atribacteria bacterium SCGC AD-561-N23 as identified according to the Kyoto Encyclopedia of Genes and Genomes (KEGG) Orthology and clusters of orthologous groups (COGs)

| Metabolic Category | Protein coding genes with COGs | Protein coding genes with KEGG Orthology |
| --- | --- | --- |
| Amino Acid Metabolism | 2 | 6 |
| Carbohydrate Metabolism | 7 | 25 |
| Lipid Metabolism | 9 | 2 |
| Nucleotide Metabolism | 2 | 3 |
| Mechanisms for foreign DNA | 4 | 1 |
| Motility | 3 | 2 |
| Replication, Transcription, and Translation | 26 | 19 |
| Ribosomal Proteins | 12 | 12 |
| Energy and conversion | 7 | 12 |
|  |  |  |
| Putative proteins, 207 proteins |  |  |

**Supplemental Table 4**. Genes of Atribacteria bacterium SCGC AD-561-N23 related to transport into and out of the cell

| Gene name | IMG ID |
| --- | --- |
| Ribose ABC transport system, periplasmic ribose-binding protein RbsB (TC 3.A.1.2.1) | 2590808876 |
| MatE, putative efflux protein | 2590808878 |
| MatE, putative efflux protein | 2590808879 |
| ABC transporter, ATP-bindin | 2590808914 |
| Substrate-specific component RibU of riboflavin ECF transporter | 2590809008 |
| large conductance mechanosensitive channel protein | 2590809085 |
| Na+-transporting methylmalonyl-CoA/oxaloacetate decarboxylase, beta subunit | 2590809090 |
| Na+-transporting oxaloacetate decarboxylase beta subunit | 2590809092 |
| LAO/AO transport system ATPase | 2590809097 |
| ABC-type uncharacterized transport system, periplasmic component | 2590809103 |
| ABC transporter permease protein | 2590809105 |
| MMPL family | 2590809107 |
| ABC transporter | 2590809113 |
| Predicted permeases | 2590809114 |

Supplemental References

Altschul, S. F., Gish, W., Miller, W., Myers, E. W. & Lipman, D. J. (1990). Basic Local Alignment Search Tool. *J. Mol. Biol.,* 215, 403-410.

Nakai, R., Abe, T., Baba, T., Imura, S., Kagoshima, H., Kanda, H.*, et al.* (2012). Microflorae of aquatic moss pillars in a freshwater lake, East Antarctica, based on fatty acid and 16S rRNA gene analyses. *Polar Biol.,* 35**,** 425-433.
